# Phase analysis of gated PET in the evaluation of mechanical ventricular synchrony: A narrative overview

**DOI:** 10.1007/s12350-019-01670-7

**Published:** 2019-03-04

**Authors:** Luis Eduardo Juarez-Orozco, Andrea Monroy-Gonzalez, Niek H. J. Prakken, Walter Noordzij, Juhani Knuuti, Robert A. deKemp, Riemer H. J. A. Slart

**Affiliations:** 1 Turku PET Centre, University of Turku and Turku University Hospital, Kiinamyllynkatu 4-8, 20520 Turku, Finland; 2 Department of Nuclear Medicine and Molecular Imaging, University Medical Center Groningen, University of Groningen, Hanzeplein 1, P.O. Box 30001, 9700 RB Groningen, The Netherlands; 3 Division of Cardiology, Department of Medicine, National Cardiac PET Centre, University of Ottawa Heart Institute (UOHI), University of Ottawa, Ottawa, ON Canada; 4 Biomedical Photonic Imaging, Technical Medical Centre, University of Twente, Enschede, The Netherlands

**Keywords:** Ventricular synchrony, phase analysis, gated PET

## Abstract

Noninvasive imaging modalities offer the possibility to dynamically evaluate cardiac motion during the cardiac cycle by means of ECG-gated acquisitions. Such motion characterization along with orientation, segmentation preprocessing, and ultimately, phase analysis, can provide quantitative estimates of ventricular mechanical synchrony. Current evidence on the role of mechanical synchrony evaluation is mainly available for echocardiography and gated single-photon emission computed tomography, but less is known about the utilization of gated positron emission tomography (PET). Although data available are sparse, there is indication that mechanical synchrony evaluation can be of diagnostic and prognostic values in patients with known or suspected coronary artery disease-related myocardial ischemia, prediction of response to cardiac resynchronization therapy, and estimation of risk for adverse cardiac events in patients’ heart failure. As such, the evaluation of mechanical ventricular synchrony through phase analysis of gated acquisitions represents a value addition to modern cardiac PET imaging modality, which warrants further research and development in the evaluation of patients with cardiovascular disease.

**Electronic supplementary material:**

The online version of this article (10.1007/s12350-019-01670-7) contains supplementary material, which is available to authorized users.

## Introduction

Beyond their capabilities to characterize myocardial architecture, perfusion, viability, and function, noninvasive imaging modalities offer the added possibility to dynamically evaluate ventricular motion during the cardiac cycle by means of ECG-gated acquisitions.^1^,^2^ Such motion characterization is achieved through sequential target detection, cavity orientation, segmentation preprocessing, and motion analysis resulting in quantitative estimates of ventricular mechanical synchrony.^3^

Currently, evidence on the evaluation of mechanical synchrony is mainly available for echocardiography, equilibrium radionuclide angiocardiography,4 and gated single-photon emission computed tomography (SPECT), while fewer reports have focused on the utilization of gated positron emission tomography (PET). The principles, parameters, and available evidence on the use of PET imaging for mechanical synchrony evaluation are summarized in this review.

## Cardiac Gated PET

PET represents a state-of-the-art modality in cardiac imaging that allows the evaluation of quantitative physiological parameters (e.g., myocardial blood flow, glucose uptake, and oxidative metabolism) determined by the selected radiotracer. The intrinsic advantages of PET in comparison to SPECT technology such as higher count rates, more physiological tracers, and increased spatial resolution provide high-quality and quantitative images that boost the diagnostic and prognostic utility at a reasonable radiation burden.

**Figure 1:**
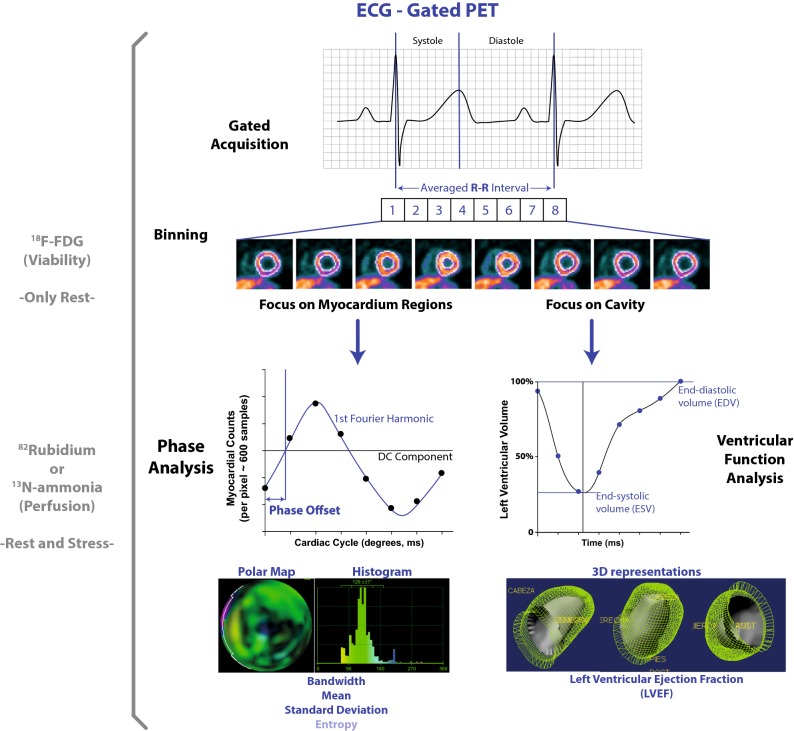
Phase and volume analyses of ECG-gated PET. DC represents the average value of mechanical contraction for a particular pixel

Current PET scanners operate with list-mode acquisitions in order to obtain adequate datasets for the reconstruction of dynamic, static, and particularly (ECG-) gated images. The latter considers the ECG signal obtained in parallel to the acquisition and tracks wall thickening and changes in the detected cavity contours throughout the averaged cardiac cycle, typically binned into 8 or 16 frames (notably, phantom research has demonstrated that 8 or 16 frames per cycle Fourier phase analysis is equally effective to detect phase delays as with 64 frames per cycle non-Fourier analysis^5^). This processing provides quantitative estimations of left-ventricular cavity volumes and consequently, the derived left ventricular ejection fraction (LVEF).^6^,^7^ Thereon, a distinctive evaluation can be performed in order to estimate parameters of ventricular synchrony of contraction through phase analysis as illustrated in Figure 1.

## Phase Analysis for Ventricular Synchrony

Phase analysis was developed originally by Chen and colleagues,^8^ and has become an interesting value-added tool in nuclear imaging. In such analysis, a large number of transmural regions in the left ventricular myocardium (500-1000) are *sampled* by evaluating the myocardial counts detected throughout the re-binned frames of the averaged cardiac cycle. These three-dimensional count distributions are analyzed using a first-harmonic Fourier (sinusoidal) function (Figure 1) for every sample of the myocardium. This allows for the measurement of the phase offset and amplitude, which provides an index of myocardial wall thickening. The phase offset shows the difference between the start-time of the first frame and the time when the sinusoidal function crosses the DC component of the myocardial counts, which represents the average value of mechanical contraction for a particular pixel. This point of convergence is interpreted as the moment of onset of the ventricular contraction for the considered sample. Finally, the collection of all phase offsets corresponding with every spatial sample can be displayed in a color-coded histogram with an *x*-axis standardized to the length of the average cardiac cycle expressed in milliseconds, periodic degrees, or a relative percentage. Moreover, it is also possible to track the onset of mechanical relaxation from a multiharmonic analysis with count-drop correction, which would correspond with the diastolic mechanical synchrony.^5^ This last approach, however, has not been significantly evaluated in PET imaging.

**Figure 2:**
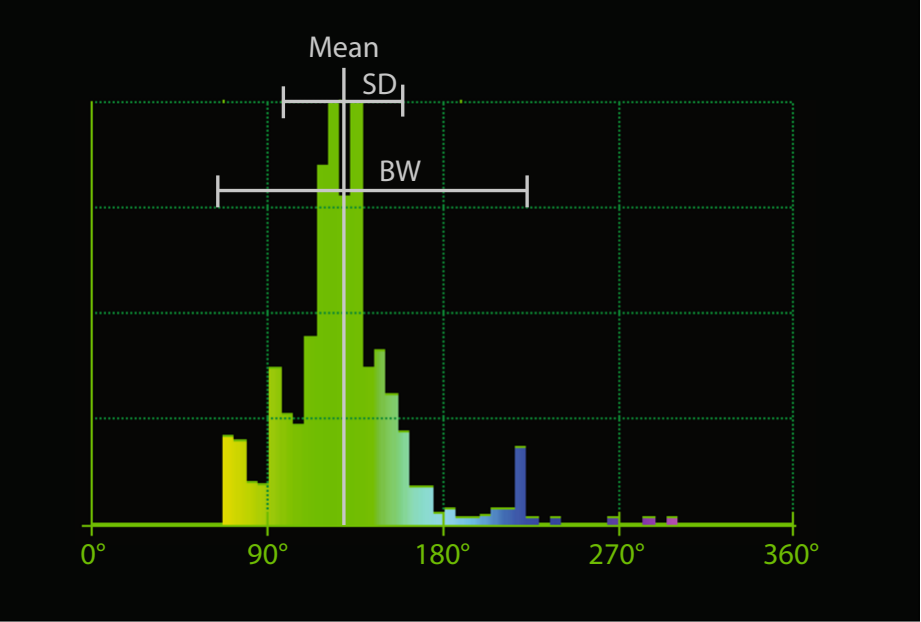
Phase histogram used to define the average onset of contraction (mean), and regional standard deviation (SD) and bandwidth (BW)

The resulting phase histogram provides several descriptive parameters of the synchronicity and uniformity of contraction of the left ventricle (see Figure 2), both as a whole or following standard segmentation procedures. Described parameters include phase mean, phase standard deviation (SD), phase bandwidth (BW = 1.96 × SD), synchrony (S) , and entropy (E).^9^ The phase mean and SD represent the average moment of phase offsets in the whole LV and the corresponding standard deviation over all myocardial samples. Phase bandwidth represents the interval where 95% of the values occur in the histogram (i.e., the range during which 95% of the ventricle initiates mechanical contraction). Entropy and Synchrony, as proposed by O’Connell et al^10^ for planar imaging, then generalized to SPECT,^11^,^12^ are slightly different metrics combining the amplitude and phase of dyssynchrony during ventricular contraction, not influenced by the histogram borders or by phase similarity.^13^

Since the average cycle is obtained over several hundreds of gated cardiac cycles (multiple R-R intervals), it is possible that phase analysis may be affected when substantial rhythm or motion disturbances are encountered (e.g., in patients with atrial fibrillation or frequent ventricular extrasystoles).^14^^-^16 Correction techniques of gating errors are therefore warranted in order to obtain robust measurements in clinical practice.^17^

## PET Ventricular Synchrony Studies

In contrast with SPECT, there is a relative paucity of publications on the feasibility, validation, average parameter values in populations of interest, and clinical utility regarding PET (dys)synchrony imaging, as evidenced in Table 1. Focus has been placed in the utility of PET synchrony assessment for the distinction of patients who may benefit from cardiac resynchronization therapy (CRT) considering that the rate of nonresponders has stabilized at around 30% of patients, as selected by ECG, LVEF, and clinical heart failure (HF) criteria following current guidelines.^15^,^18^ In the setting of CAD, the link between myocardial ischemia and mechanical synchrony has been studied primarily under the working assumption that myocardial blood flow (the quantitative perfusion feature offered by PET but not SPECT imaging) may represent a determinant in the status of ventricular mechanical synchrony and its response during pharmacological stress (vide infra).

**Table 1:** PET studies on ventricular synchrony

Study	Year	Clinical setting	Aim	N	Population	PET Tracer	Software	Synchrony parameters studied
Van Tosh^22^	2017	Known or Suspected CAD	To evaluate MBF in patients with rest dyssynchrony depending on their synchrony improvement or deterioration during stress	195	53% CAD, 18% HF	^82^Rb	ECTb	BW
Juarez-Orozco^19^	2016	Known or Suspected CAD	To test MFR and sMBF as predictors of mechanical synchrony	248	CAD	^13^N-NH_3_	QPS	BW SD E
Kerrigan^23^	2015	Suspected CAD	Case report for acute stress dyssynchrony due to myocardial ischemia	1	CAD	^82^Rb	4DM	Mean SD
Lehner^27^	2013	CRT response prediction	To evaluate if amount of viable and dyssynchronous myocardium predicts CRT response	19	HF with DCM or ICM	^18^F-FDG	QPS	BW Mean SD E
Wang^41^	2013	Known CAD	To compare FDG-PET to SPECT synchrony assessment in patients with CAD	100	CAD	^18^F-FDG	QPS	BW SD
AlJaroudi^42^	2012	HF of ischemic origin	Evaluate prognostic value of dyssynchrony for survival in CABG vs. medical therapy	486	HF, CAD, and narrow QRS	^82^Rb	4DM	SD
AlJaroudi^21^	2012	Known CAD	Evaluate the effect of prior CABG and paradoxical septal motion on dyssynchrony	568	HF	^82^Rb	4DM	SD
AlJaroudi^16^	2012	Normal patients and HF patients	Evaluate differences between rest and stress synchrony in patient with normal perfusion	217	Normal perfusion, with high and low LVEF, narrow QRS	^82^Rb	4DM	SD
AlJaroudi^29^	2012	HF of ischemic origin	Evaluate stress induced dyssynchrony, its predictors, and its prognostic value	489	HF, ICM, narrow QRS	^82^Rb	4DM	SD SD change
Pazhenkottil^43^	2011	HF of ischemic origin	Compare BW and SD between SPECT-perfusion and PET-viability imaging	30	HF, ICM	^18^F-FDG	ECTb	BW SD
Cooke^30^	2011	Normal patients and LBBB patients	Develop normal synchrony values for rest and stress PET imaging and compare the values with those of patients with LBBB	63	Low Likelihood patients and patients with LBBB	^82^Rb	ECTb	Rest and stress: BW Mean SD
Uebleis^13^	2011	CRT response prediction	Retrospectively distinguish responders by scar burden, persistent dyssynchrony and misplacement of CRT leads	14	HF with CRT	^18^F-FDG	QPS	BW SD E

*BW*, bandwidth; *CAD*, coronary artery disease; *CRT*, cardiac resynchronization therapy; *DCM*, dilated cardiomyopathy; *E*, entropy; *ECTb*, Emory Cardiac Toolbox; *HF*, heart failure; *ICM*, ischemic cardiomyopathy; *LVEF*, left ventricular ejection fraction; *SD*, standard deviation

A large number of published reports on mechanical ventricular synchrony evaluated with PET have utilized ^18^F-FDG and ^82^Rb as viability and perfusion radiotracers, respectively. In fact, only one study has evaluated correlates and determinants of synchrony measurements from ^13^N-ammonia PET perfusion data,^19^ while no study has utilized ^15^O-water for such evaluation.

### Predictors of PET Ventricular Synchrony

A number of variables have been proposed to associate with mechanical dyssynchrony in retrospective studies such as QRS duration (as the surrogate for electrical dyssynchrony), intraventricular conduction delay (as seen in patients with left bundle branch block [LBBB]) and LVEF.^20^ With PET imaging particularly, sex, age, the presence of type-2 diabetes mellitus, and impaired quantitative stress myocardial perfusion have demonstrated an independent effect on a constellation of PET-derived ventricular function parameters that included Entropy^19^ in patients with known or suspected CAD. Additionally, in patients with HF, the degree of ventricular remodeling, perfusion defect size, atrial fibrillation, BMI and LVEF have been reported as independent predictors of mechanical synchrony (evaluated using phase SD).^21^ These data underline how a different but overlapping range of relevant predictors of dyssynchrony may be considered according to the clinical scenario.

### Role in Coronary Artery Disease

**Figure 3:**
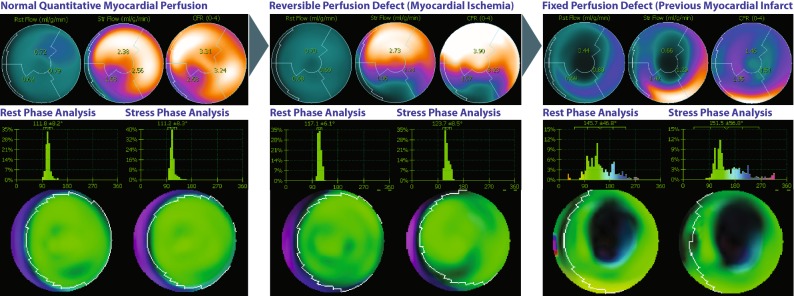
Phase synchrony evaluation in patients along the spectrum of ischemic heart disease (*left panel:* normal perfusion, *middle panel*: severe inferoseptal myocardial ischemia, and *right panel*: with previous anteroapical transmural myocardial infarction and moderate residual ischemia). Delayed onset of contraction is typically observed in the regions of ischemia and infarction

A parallel working concept in the field of cardiac PET deals with the relationship between myocardial ischemia and ventricular synchrony.^19^,^22^,^23^ Notably, the characterization of this interaction seems to be suitable for the application of PET due to the fact that myocardial perfusion studies are typically acquired during conditions of peak-stress (in contrast to the poststress evaluation with SPECT imaging). Phase synchrony evaluation has therefore been proposed as a marker in the detection of myocardial stunning and ischemia-induced dyssynchrony.^24^ Specifically, synchrony differences in between rest and stress acquisitions have been demonstrated. Synchrony indices have been found to be lower during peak stress in patients with normal myocardial perfusion possibly due to improved contractility. Interestingly, these differences have been described in patients with normal and low LVEF.^16^ Figure 3 depicts representative examples of PET-measured ventricular synchrony along the continuum of ischemic heart disease.

Although SPECT studies have aimed to better characterize the phenomenon,^25^ it is still unknown how the perfusion-synchrony relation may operate at the regional level with the utilization of PET. Moreover, it is also unclear to what extent may the evaluation of PET synchrony improve the detection of significant CAD beyond other robust functional variables such as LVEF.

### Role in Heart Failure and CRT Response Prediction

In patients with HF who may ultimately attract criteria for the indication of CRT^18^ (i.e., LVEF ≤ 35%, QRS > 150 ms, and NYHA functional classification ≥ II), there is a notion that a proportion of effective response to CRT could be explained by an underlying substrate of mechanical dyssynchrony (which is not evaluated in formal selection of CRT recipients, but only partially captured by the electrical synchrony criteria). Suggested variables have been proposed to associate with adequate response to the therapy such as location and extent of PET-defined myocardial viability, extent of scarring and optimal lead placement, LV volumes, and indeed, ventricular mechanical dyssynchrony.^13^,^26^,^27^ The challenge to effectively integrate every relevant PET-derived variable to refine CRT patient selection in a medium-to-large scale study remains ubiquitous.

### Prognostic Value of PET Synchrony Evaluation

Only a handful of studies performed with PET have addressed the potential prognostic value of mechanical synchrony. The results of this very discrete body of evidence are inclined to be in favor of a discernible independent hazard ratio of synchrony measures as predictors of all-cause mortality in patients with ischemic cardiomyopathy,^28^ and patients with HF and a narrow QRS (1.16 [1.03, 1.30] per 10° increase in SD and 1.19 [1.01, 1.38] per 10° increase in SD response).^21^,^29^

## Reference Values

Table 2 outlines the reports that have suggested reference values (i.e., normal values and cutoff points for distinguishing from pathological populations) in the evaluation of mechanical synchrony with PET and SPECT (selected for comparison). In fact, when analyzing available reports, it is noticeable how assumptions of robustness, and in some cases of normal values, have been directly translated from SPECT studies. Although it is true that PET could be understood as a refined version of SPECT imaging due to lower noise, higher tracer counts, lower radiation burden, and improved spatial resolution,^15^ it is of great relevance to characterize how these factors may influence the estimation of normal and pathological synchrony values in order to promote the utilization of PET synchrony evaluation with different protocols and software packages. In this sense, the study by Cooke et al complementarily compared their estimates to those suggested in previous SPECT studies concluding that very likely BW and SD are robust and reproducible measures of synchrony across stressors, physiologic states, acquisitions, reconstruction methodologies, and processing algorithms.^30^ Further in general, factors like age, LVEF, and heart rate may affect the dyssynchrony results. SPECT studies have reported variability in volumes and ejection fraction by different software.^31^,^32^ Also, larger values of phase bandwidth, phase SD, and entropy have been reported for men compared to women in SPECT studies.^33^,^34^ These assumptions, however, should be utilized with caution when evaluating PET-derived synchrony.

**Table 2:** Reference values and discrimination cutoffs

Technique	Study	Year	Sample	Software	Normal values	Cutoff points
SPECT	Okuda^35^	2017	122 normal perfusion and LVEF, 34 with suspected dyssynchrony	CardioREPO 4DM ECTb QGS	BW = 38.4° ± 10.4 SD = 9.7° ± 2.8 E = 41.9% ± 6.2	BW = 24-42° SD = 8.6°-15.3° E = 31-48%
PET	AlJaroudi^16^	2012	91 normal perfusion and LVEF, 126 with low LVEF	4DM	rSD = 16.8° ± 7.8 sSD = 12.4° ± 3.7	SD = 20°
PET	Cooke^30^	2011	40 low likelihood of CAD (20 men and 20 women) and 23 with LBBB (10 men and 13 women)	ECTb	Men rBW = 50.8° ± 18.7 sBW = 38.1° ± 13.3 rSD = 22.7° ± 13.2 sSD = 15.0° ± 7.0 Women rBW = 44.4° ± 44.9 sBW = 32.0° ± 13.5 rSD = 16.6° ± 14.3 sSD = 13.2° ± 7.7	Men rBW = 49° sBW = 52° rSD = 22.1° sSD = 26.1° Women rBW = 50° sBW = 33° rSD = 15.7° sSD = 13.7°
SPECT	Boogers^44^	2009	40 HF with CRT indication (24 CRT responders and 16 nonresponders)	QGS	-	BW = 72.5° SD = 19.6°
SPECT	Henneman^45^	2007	42 HF with CRT indication (30 CRT responders and 12 nonresponders)	ECTb	-	BW = 135° SD = 43°
SPECT	Chen^8^	2005	90 low likelihood of CAD (45 men and 45 women)	ECTb	Men BW = 38.7° ± 11.8 SD = 14.2° ± 5.1 Women BW = 30.6° ± 9.6 SD = 11.8° ± 5.2	Men BW = 38.7° ± 11.8 SD = 14.2° ± 5.1 Women BW = 30.6° ± 9.6 SD = 11.8° ± 5.2

*BW*, bandwidth; *CAD,* coronary artery disease; *CRT*, cardiac resynchronization therapy; *E*, entropy; *ECTb*, Emory Cardiac Toolbox; *HF*, heart failure; *LBBB*, left bundle branch block; *LVEF*, left ventricular ejection fraction; *r*, rest; *s*, stress; *SD*, standard deviation

Another factor of interest is the availability of several commercial software packages that offer phase analysis. Overall, phase analysis has been implemented in the Emory Cardiac Toolbox 4DM and QGS software. Variability across packages has recently been addressed by Okuda et al,^35^ but only in the case of SPECT acquisitions. Cross-validation efforts in synchrony evaluation with PET are therefore warranted to enable comparison of measured values between imaging centers using different software programs.

In summary, ventricular mechanical synchrony as measured by PET imaging may be of value in the evaluation of patients with suspected myocardial ischemia leading to myocardial stunning and in patients with HF with an indication for CRT due to the suspected substrate of mechanical dyssynchrony. At the same time, it is likely that PET synchrony evaluation may hold prognostic values in patients with HF and in patients with CAD, in particular with multivessel disease BW of which and the SD of the phase after exercise are significantly increased. In addition, phase analysis is able to detect the LV mechanical dyssynchrony due to the vasomotion changes associated with occult atherosclerosis in patients with normal coronary angiography findings. Whether PET-measured synchrony can offer diagnostic value beyond or at an earlier stage than mainstream functional parameters, may serve as a tool for refining selection of CRT recipients, and should be incorporated in the clinical exercise of risk stratification, remains to be elucidated. The application of PET synchrony evaluation together with the evaluation of myocardial scar (fibrosis) has the potential to improve selection for access to CRT in those patients most likely to improve the clinical effectiveness and cost effectiveness of CRT for heart failure.

Notably, the intrinsic advantages of PET, including its wide range of physiological radiotracers available and its full quantitative capabilities, set the ground for the value addition to the phase analysis of ventricular synchrony in establishing the so-called “one-stop shop”^15^ in which perfusion or viability, scar location, and extent, ventricular volumes, and function (both systolic and diastolic), and synchrony^36^ can be simultaneously evaluated. Moreover, comprehensive imaging can be boosted through the utilization of currently available hybrid equipment (PET/CT and PET/MR) that allows for complementary anatomic information (e.g., epicardial fat, calcium score, and venous system structure) to be obtained within the same imaging session. Cardiac MR (CMR) is, in addition to PET, is expected to provide—partly confirming, partly complementary—tissue-specific anatomic (fiber, fat, muscle, and blood) and pathophysiological (edema, infarction, microvascular obstruction, and tumor) information, and could add tissue strain data which can be used as a measure of cardiac synchrony to complete a disease-specific cardiac model, as was recently reported for a carotid plaque inflammation model using MR-PET/CT,^37^ and in a cardiac sarcoidosis model using CMR, PET, and ultrasound,^38^ and in a hypertrophic cardiomyopathy (HCM)-phenotype model using CMR, PET, and ultrasound.^39^ The recently published joint position statement of the ESCR and EANM also states application of CMR-PET is feasible, robust, and promising.^40^ We therefore expect cardiac gated CMR-PET to provide a new model to help understand cardiac synchrony in future studies.

## New Knowledge Gained

Evaluation of PET ventricular mechanical synchrony has arguably emerged as an extrapolation of prior phase analysis using SPECT imaging. As such, there are variations in reference values, and extensive evidence on its utility for the evaluation of ventricular dysfunction with diagnostic and prognostic purposes as well as for better selection of CRT recipients is slowly emerging.

## Conclusion

The evaluation of mechanical ventricular synchrony through phase analysis of gated acquisitions represents a value addition to modern cardiac PET imaging. Cardiac PET synchrony may be useful in the assessment of patients with CAD, in the evaluation of prognosis in patients with cardiac dysfunction, and in the optimization of patient selection for advanced therapies such as CRT.

## Electronic supplementary material

Below is the link to the electronic supplementary material.

Supplementary material 1 (PPTX 2062 kb)

## Abbreviations

BW: Bandwidth
CAD: Coronary artery disease
CMR: Cardiac magnetic resonance
CRT: Cardiac resynchronization therapy
E: Entropy
HF: Heart failure
LVEF: Left ventricular ejection fraction
MBF: Myocardial blood flow
MFR: Myocardial flow reserve
PET: Positron emission tomography
SD: Standard deviation
SPECT: Single-photon emission computed tomography
SRS: Summed rest score
