# Supplementary material for: Phase analysis of gated PET in the evaluation of mechanical ventricular synchrony: A narrative overview
Source: J Nucl Cardiol. 2019 Mar 4;26(6):1904–13. doi: 10.1007/s12350-019-01670-7 (PMC6908565; doi:10.1007/s12350-019-01670-7)
Supplement: Supplementary file 1 — Supplementary material 1 (PPTX 2062 kb) [file 12350_2019_1670_MOESM1_ESM.pptx]

## Slide 1
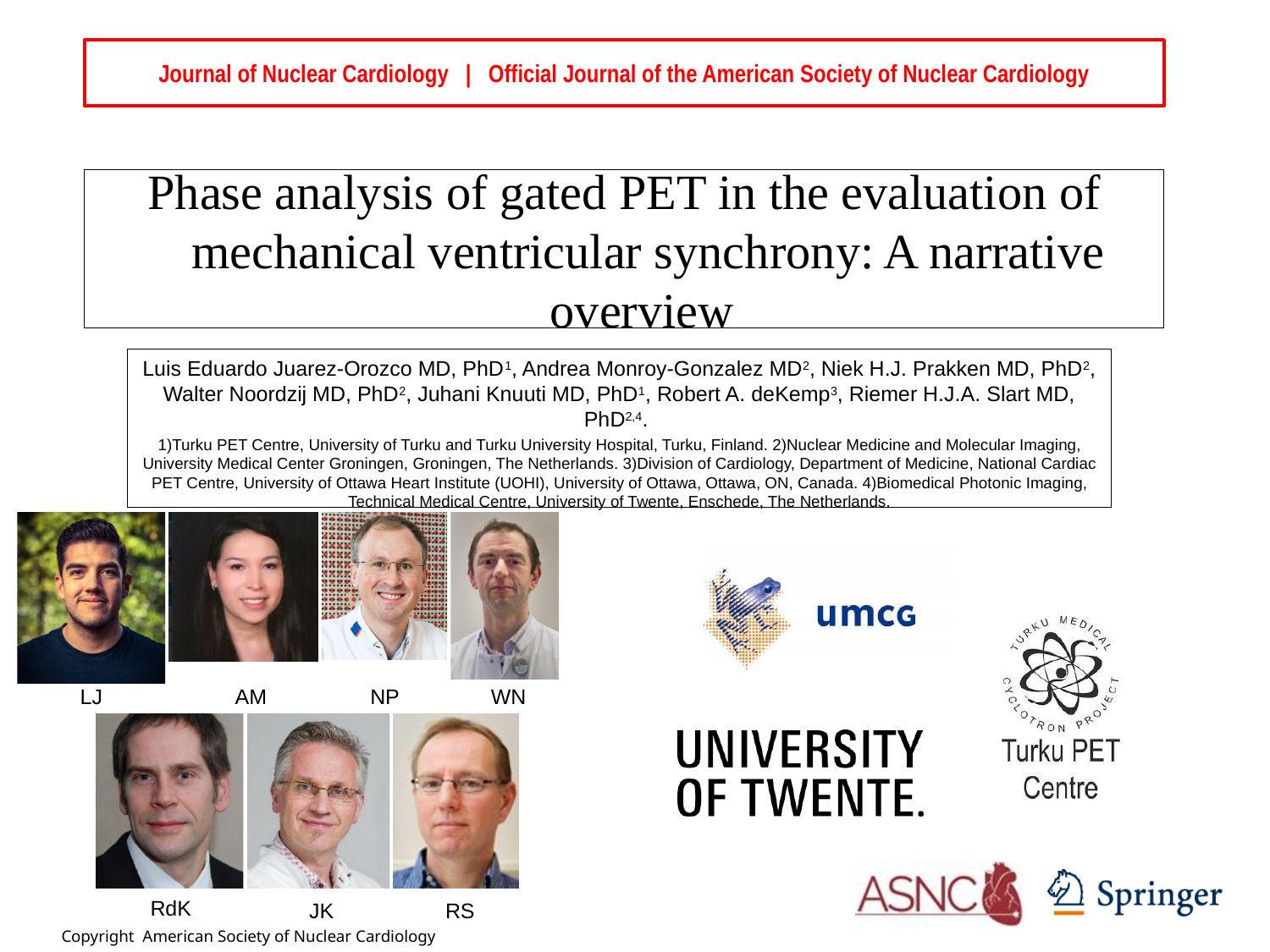

Journal of Nuclear Cardiology | Official Journal of the American Society of Nuclear Cardiology
# Phase analysis of gated PET in the evaluation of mechanical ventricular synchrony: A narrative overview
Luis Eduardo Juarez-Orozco MD, PhD1, Andrea Monroy-Gonzalez MD2, Niek H.J. Prakken MD, PhD2, Walter Noordzij MD, PhD2, Juhani Knuuti MD, PhD1, Robert A. deKemp3, Riemer H.J.A. Slart MD, PhD2,4.
1)Turku PET Centre, University of Turku and Turku University Hospital, Turku, Finland. 2)Nuclear Medicine and Molecular Imaging, University Medical Center Groningen, Groningen, The Netherlands. 3)Division of Cardiology, Department of Medicine, National Cardiac PET Centre, University of Ottawa Heart Institute (UOHI), University of Ottawa, Ottawa, ON, Canada. 4)Biomedical Photonic Imaging, Technical Medical Centre, University of Twente, Enschede, The Netherlands.
LJ
AM
NP
WN
RdK
JK
RS
Copyright American Society of Nuclear Cardiology

## Slide 2
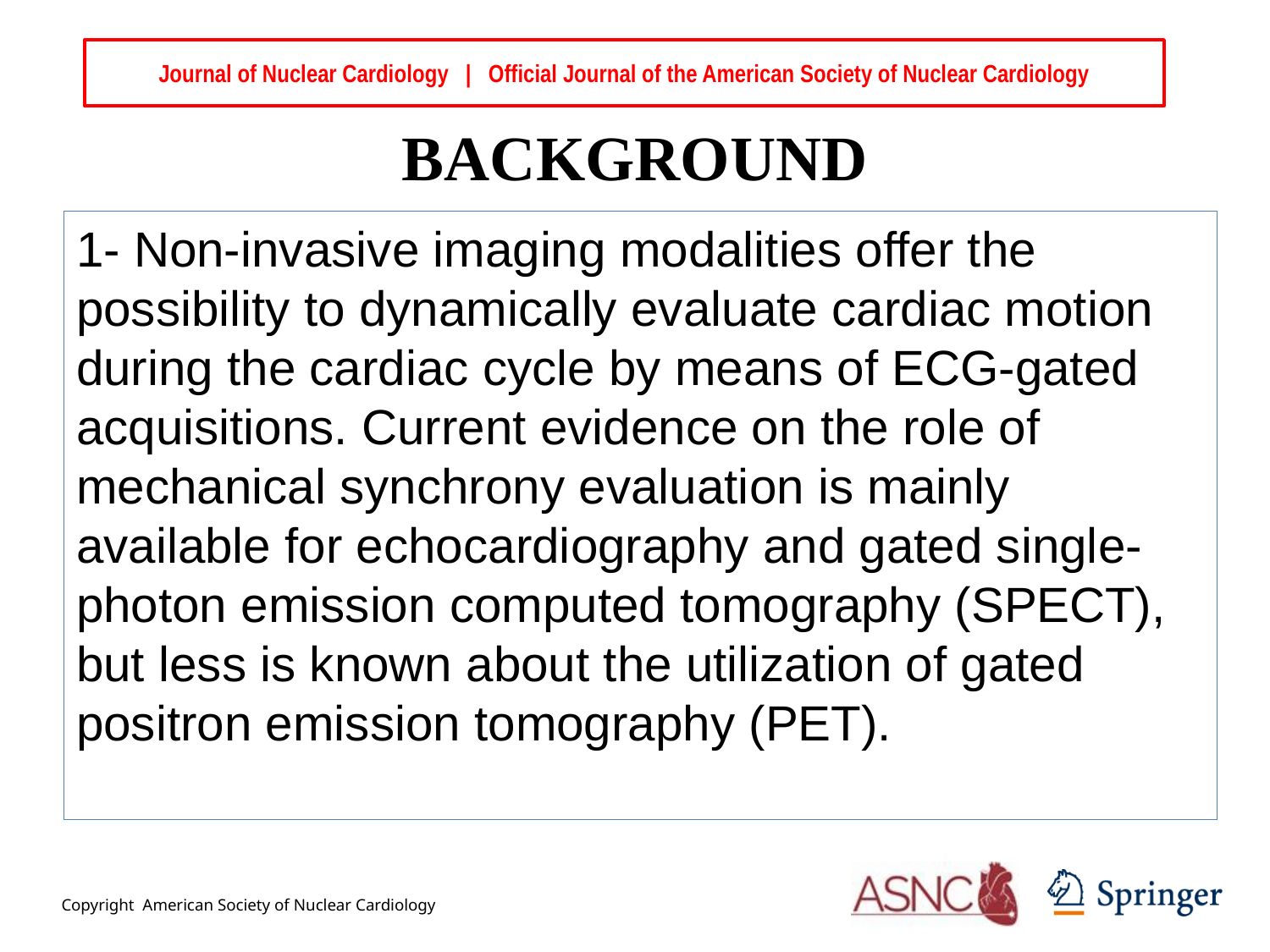

Journal of Nuclear Cardiology | Official Journal of the American Society of Nuclear Cardiology
# BACKGROUND
1- Non-invasive imaging modalities offer the possibility to dynamically evaluate cardiac motion during the cardiac cycle by means of ECG-gated acquisitions. Current evidence on the role of mechanical synchrony evaluation is mainly available for echocardiography and gated single-photon emission computed tomography (SPECT), but less is known about the utilization of gated positron emission tomography (PET).
Copyright American Society of Nuclear Cardiology

## Slide 3
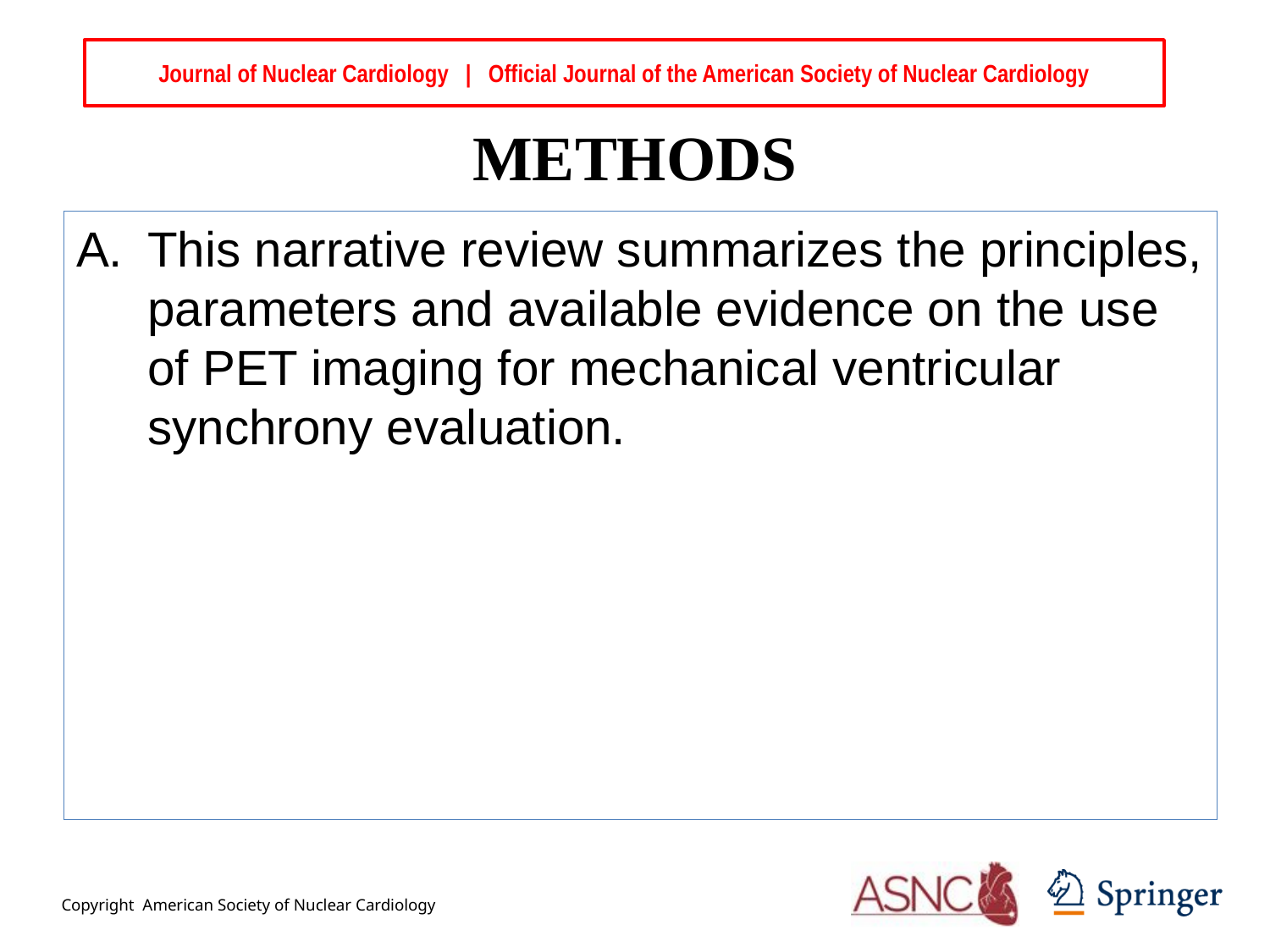

Journal of Nuclear Cardiology | Official Journal of the American Society of Nuclear Cardiology
# METHODS
This narrative review summarizes the principles, parameters and available evidence on the use of PET imaging for mechanical ventricular synchrony evaluation.
Copyright American Society of Nuclear Cardiology

## Slide 4
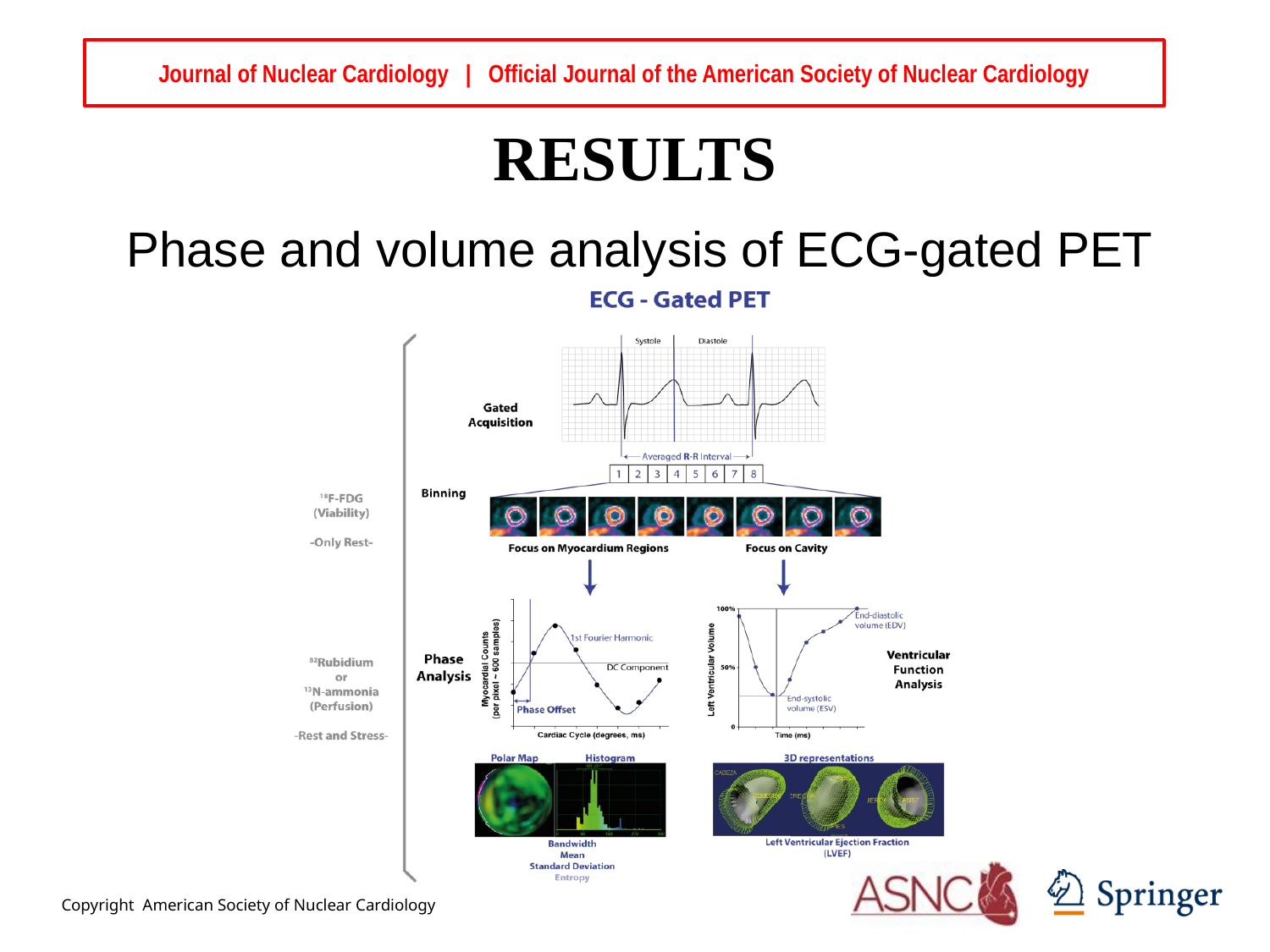

Journal of Nuclear Cardiology | Official Journal of the American Society of Nuclear Cardiology
# RESULTS
Phase and volume analysis of ECG-gated PET
Copyright American Society of Nuclear Cardiology

## Slide 5
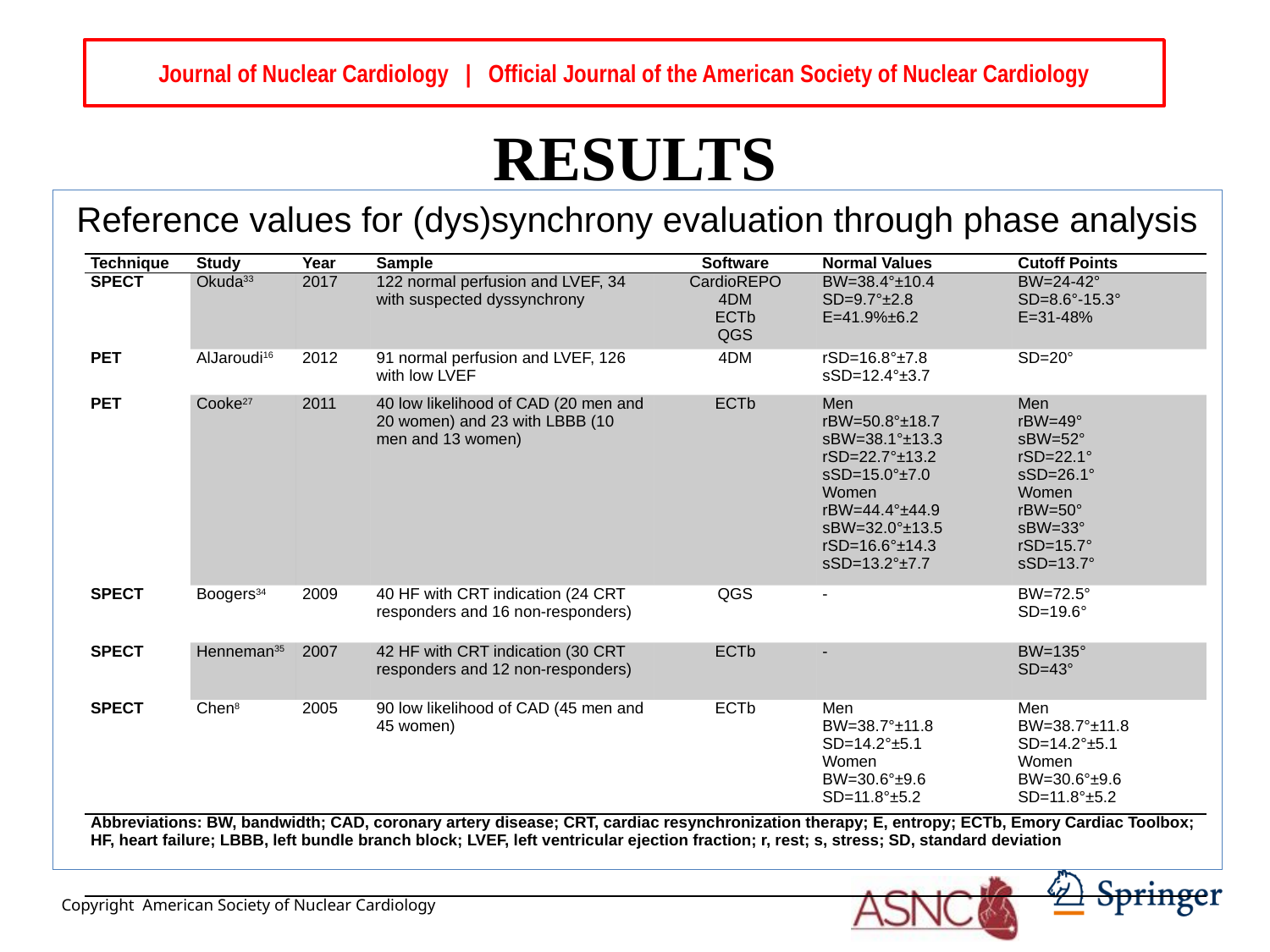

Journal of Nuclear Cardiology | Official Journal of the American Society of Nuclear Cardiology
# RESULTS
Reference values for (dys)synchrony evaluation through phase analysis
| Technique | Study | Year | Sample | Software | Normal Values | Cutoff Points |
| --- | --- | --- | --- | --- | --- | --- |
| SPECT | Okuda33 | 2017 | 122 normal perfusion and LVEF, 34 with suspected dyssynchrony | CardioREPO 4DM ECTb QGS | BW=38.4°±10.4 SD=9.7°±2.8 E=41.9%±6.2 | BW=24-42° SD=8.6°-15.3° E=31-48% |
| PET | AlJaroudi16 | 2012 | 91 normal perfusion and LVEF, 126 with low LVEF | 4DM | rSD=16.8°±7.8 sSD=12.4°±3.7 | SD=20° |
| PET | Cooke27 | 2011 | 40 low likelihood of CAD (20 men and 20 women) and 23 with LBBB (10 men and 13 women) | ECTb | Men rBW=50.8°±18.7 sBW=38.1°±13.3 rSD=22.7°±13.2 sSD=15.0°±7.0 Women rBW=44.4°±44.9 sBW=32.0°±13.5 rSD=16.6°±14.3 sSD=13.2°±7.7 | Men rBW=49° sBW=52° rSD=22.1° sSD=26.1° Women rBW=50° sBW=33° rSD=15.7° sSD=13.7° |
| SPECT | Boogers34 | 2009 | 40 HF with CRT indication (24 CRT responders and 16 non-responders) | QGS | - | BW=72.5° SD=19.6° |
| SPECT | Henneman35 | 2007 | 42 HF with CRT indication (30 CRT responders and 12 non-responders) | ECTb | - | BW=135° SD=43° |
| SPECT | Chen8 | 2005 | 90 low likelihood of CAD (45 men and 45 women) | ECTb | Men BW=38.7°±11.8 SD=14.2°±5.1 Women BW=30.6°±9.6 SD=11.8°±5.2 | Men BW=38.7°±11.8 SD=14.2°±5.1 Women BW=30.6°±9.6 SD=11.8°±5.2 |
| Abbreviations: BW, bandwidth; CAD, coronary artery disease; CRT, cardiac resynchronization therapy; E, entropy; ECTb, Emory Cardiac Toolbox; HF, heart failure; LBBB, left bundle branch block; LVEF, left ventricular ejection fraction; r, rest; s, stress; SD, standard deviation | | | | | | |
Copyright American Society of Nuclear Cardiology

## Slide 6
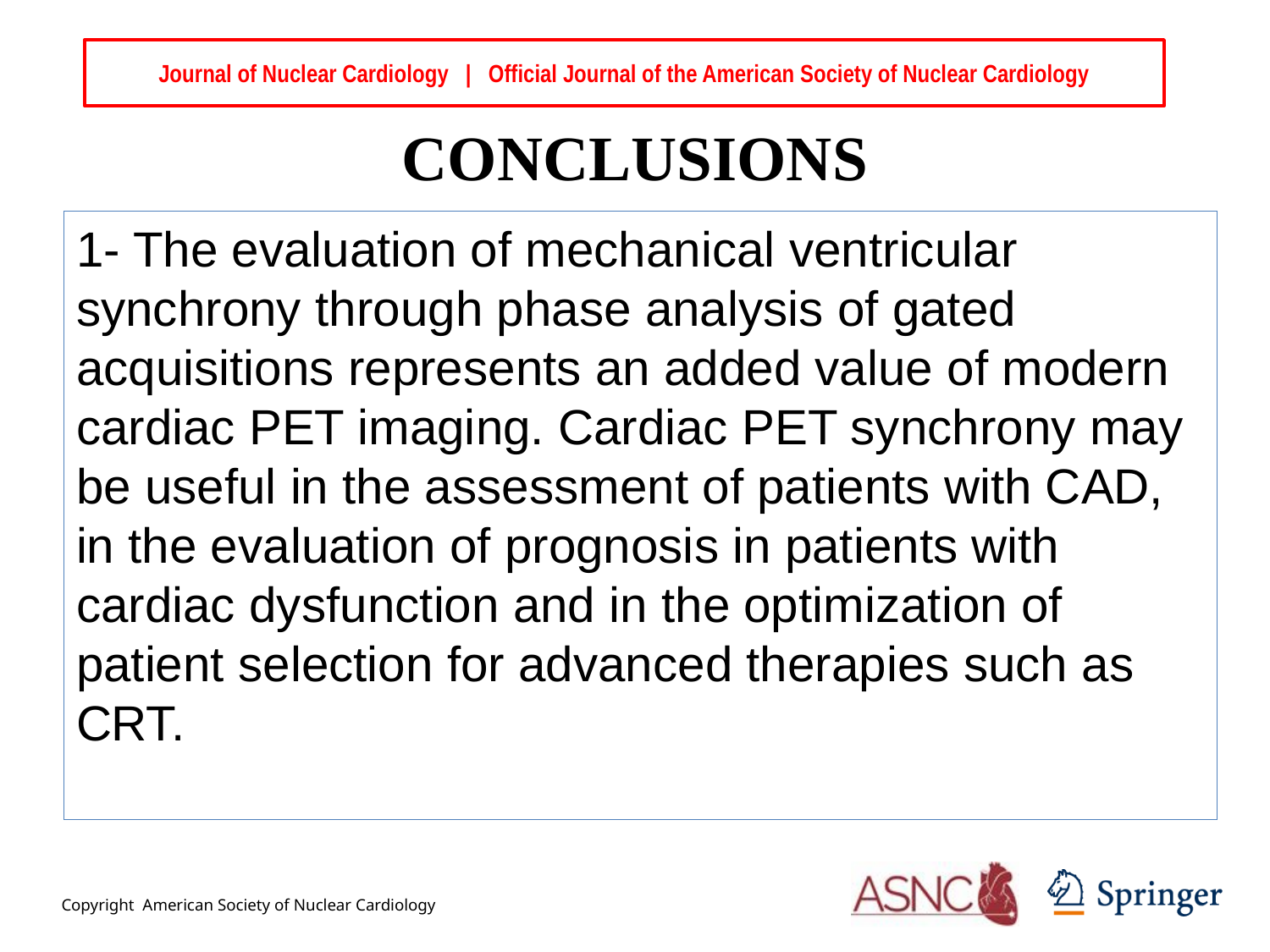

Journal of Nuclear Cardiology | Official Journal of the American Society of Nuclear Cardiology
# CONCLUSIONS
1- The evaluation of mechanical ventricular synchrony through phase analysis of gated acquisitions represents an added value of modern cardiac PET imaging. Cardiac PET synchrony may be useful in the assessment of patients with CAD, in the evaluation of prognosis in patients with cardiac dysfunction and in the optimization of patient selection for advanced therapies such as CRT.
Copyright American Society of Nuclear Cardiology
